# Exploring the correlates of frequency of Tai Chi Chuan participation among Chinese adults in an urban context

**DOI:** 10.3389/fpsyg.2025.1612546

**Published:** 2025-11-17

**Authors:** Yizhen Chao, Yaqing Wang, Yongtao Yu

**Affiliations:** 1 The Wushu Association of Dengzhou City, Dengzhou, Henan, China; 2 School of Physical Education, Leshan Normal University, Leshan, Sichuan, China

**Keywords:** Tai Chi Chuan, sports participation, physical activity, correlates, adults, China

## Abstract

**Background:**

Tai Chi Chuan (TCC) has achieved global recognition and was inscribed on the UNESCO Representative List of Intangible Cultural Heritage of Humanity in 2020. However, a significant gap remains in research examining the correlates of TCC participation. This study aims to address this gap by analyzing data from a sample of adult TCC practitioners in urban parks in Zhengzhou City.

**Methods:**

This study collected data from 998 Chinese adults practicing TCC in public parks in Zhengzhou City, Henan Province, using face-to-face questionnaire interviews. Data analysis involved descriptive statistics, univariate analysis, and backward stepwise ordinal regression modeling.

**Results:**

Twelve independent variables were found to be significantly associated with the frequency of TCC participation: age, mental problems, health problems, investment, alternate spaces, distance, awards, health motivation, partners, gender, nature of labor, and migration. The remaining variables showed no significant association with participation frequency.

**Conclusion:**

This study identified key determinants influencing the frequency of TCC participation among Chinese adults. The research findings provide important evidence for developing intervention strategies aimed at increasing participation frequency.

## 1 Introduction

As an integral part of traditional Chinese culture, Tai Chi Chuan (TCC) has achieved widespread global dissemination. In 2020, UNESCO inscribed TCC on the Representative List of the Intangible Cultural Heritage of Humanity, further enhancing its international recognition (United Nations Educational and Organization, 2020). TCC is a moderate-intensity physical activity characterized by weight shifting, body rotation, and semi-squat movements. It can be practiced individually or in groups. Compared with high- intensity sports such as basketball and football, TCC emphasizes slow and gentle movements, making it not only beneficial for physical health but also widely regarded as an effective method of disease prevention and wellness (Lan et al., 2002; Jiménez-Martín et al., 2013; Yang et al., 2015). particularly, it is suitable for middle-aged and older adults, contributing to its broad acceptability and adaptability among adult populations.

In China, the government has strongly supported the promotion of TCC. The National Fitness Guidelines (2017) and the Healthy China Initiative (2019) both recommend practicing TCC at least three times per week to achieve basic fitness benefits (Healthy China Action Promotion Committee, 2019; General Administration of Sport of the People’s Republic of China, 2017). However, despite these policy endorsements and guidelines, insufficient participation frequency may limit the associated health gains. Thus, it is essential to investigate the factors influencing TCC participation frequency to inform effective intervention strategies and facilitate widespread adoption. Although an increasing number of studies have examined TCC, most focus on experimental or clinical designs exploring its physical and mental health benefits (Wang et al., 2014; Huston and McFarlane, 2016). While such research underscores the health value of TCC, it tends to overlook behavioral aspects—specifically, whether individuals participate, how frequently, and for how long. The lack of behavioral research hinders a deeper understanding of the potential factors influencing TCC participation.

The empirical studies of TCC participation frequency research targeting Chinese adults and the relationships between individual characteristics (e.g., age, gender, and education level) remain scarce. Consequently, we lack sufficient insight into which population segments are more likely to engage in TCC frequently, making it difficult to develop targeted interventions for low-frequency participants. To address this issue, the study aims to investigate the correlates of TCC participation frequency among Chinese adults, identify key influencing factors, and support the development of targeted behavioral interventions. Such efforts can not only enhance individual engagement but also promote broader dissemination and sustainable development of TCC practice.

Although existing literature has extensively examined the correlates of general sports participation (GSP), most studies consider diverse physical activities without distinguishing among specific sport types (Crossman et al., 2024; Bauman et al., 2012; Zasimova, 2022; Borgers et al., 2016; Deelen et al., 2018; Oliveira-Brochado et al., 2017; Amornsriwatanakul et al., 2023; Eime et al., 2015; Wicker et al., 2013; Hallmann et al., 2012; Chao et al., 2025). Although TCC possesses unique characteristics as a form of physical activity (World Health Organization, 2019), it is nonetheless expected to be influenced by similar factors associated with GSP, such as age and gender. Based on its distinct movement patterns and prior literature, this study hypothesizes that factors associated with GSP—such as age and gender—are also relevant to TCC participation, albeit with potentially different effect magnitudes.

To ensure comprehensive and systematic analysis, this study incorporates a wide range of potential correlates previously shown to be significantly associated with GSP, and examines their relationship with TCC participation frequency among Chinese adults. The core contribution of this study lies in filling the current research gap on the behavioral correlates of TCC participation, particularly within adult populations in China. By identifying the key determinants of participation frequency, the findings can offer empirical support for policymakers and intervention designers, help clarify strategic priorities, and guide the development of more targeted programs aimed at increasing physical activity levels among low-frequency participants. In turn, this may improve overall participation outcomes and enhance physical and mental well-being. Furthermore, given TCC’s broad public appeal and well-documented health benefits, this study contributes to the overall promotion of physical activity among Chinese adults and supports the national goal of improving public health. It provides theoretical and practical guidance for achieving the Healthy China 2030 initiative ‘s objective of nationwide fitness.

The remainder of this paper is structured as follows. The next section outlines the study’s methodology, including data sources, survey context, variable measurement, and the models employed for analysis. This is followed by a presentation of results, including descriptive statistics and model estimates. The subsequent section provides a detailed discussion and interpretation of the findings. Finally, the paper concludes by summarizing key insights and outlining limitations and future research directions.

## 2 Methods

### 2.1 Data

This study employed a cross-sectional design and used a self-reported questionnaire to collect data, as it was not feasible to obtain objective longitudinal data on participants’ TCC practice. Data collection was conducted through face-to-face interviews by professionally trained surveyors, using a standardized and pre-designed questionnaire. All interviews were voluntary. Prior to participation, researchers clearly explained the purpose of the study and assured participants of confidentiality. Informed verbal consent was obtained from all respondents. The study strictly adhered to international ethical standards and received approval from the Research Ethics Committee of Leshan Normal University. Participants were selected using random sampling from major parks located in six administrative districts of Zhengzhou City (Zhongyuan, Erqi, Guancheng, Huiji, Jinshui, and Shangjie Districts). As the birthplace of TCC, Henan Province—and Zhengzhou City as its capital—has a deep-rooted cultural connection to TCC. Public parks in China are key venues for adults to engage in physical activity (National Physical Fitness Monitoring Center of the People’s Republic of China, 2021), and prior field observations confirmed a substantial presence of TCC practitioners in the selected locations, ensuring both the representativeness and adequacy of the sample. Data collection was carried out between May and November 2023, during early morning hours (6:00 to 7:30 a.m.) to avoid conflicts with participants’ work schedules. Before initiating contact, surveyors first observed whether individuals were actively practicing TCC. If confirmed, participants were approached during breaks or after the session to inquire about their willingness to participate. Once informed consent was obtained and adult status confirmed, the interview commenced. If participants encountered difficulties understanding any questions, interviewers provided necessary clarifications to ensure accurate responses. Participants retained the right to withdraw or decline to answer at any point during the process. To enhance the validity and comprehensibility of the questionnaire, a pilot study was conducted prior to formal data collection. In two parks in Zhengzhou, a subset of participants completed the questionnaire in the presence of interviewers, who observed their understanding of each item. Based on participant feedback, the questionnaire was revised and finalized accordingly. During the data entry phase, a dual-entry system combined with manual verification was used to minimize potential input errors. The planned sample size was 1,200 adults aged 18 and above. Ultimately, 998 valid questionnaires were collected, resulting in a response rate of 83%. Data from 202 participants who failed to complete the questionnaire were excluded from the final analysis.

### 2.2 Measures

The questionnaire used in this study was designed to collect data related to participants’ frequency of TCC practice. Given that TCC is a form of physical activity, the questionnaire was developed with reference to established literature on GSP. It incorporated variables that have been empirically validated as significantly associated with GSP. The questionnaire encompassed a wide range of factors, including sociodemographic characteristics and environmental contexts, aiming to comprehensively capture the correlates of TCC participation frequency.

#### 2.2.1 Measurement of TCC participation frequency

To assess the frequency of TCC participation, respondents were asked to indicate the average number of times per week they had practiced TCC over the past month. To facilitate analysis of participation trends across population groups with different characteristics, the outcome variable—TCC participation frequency—was constructed as an ordinal categorical variable. Specifically, participation frequency was categorized into four ascending levels: (1) 1–2 times per week, (2) 3–4 times per week, (3) 5–6 times per week, and (4) 7 or more times per week. All 998 valid responses provided complete data on the average weekly frequency of TCC participation.

#### 2.2.2 Measurement of independent variables

In this study, we drew upon existing literature on GSP to select a comprehensive set of independent variables. These variables have been consistently identified as significant correlates of physical activity in prior research (Crossman et al., 2024; Bauman et al., 2012; Zasimova, 2022; Borgers et al., 2016; Deelen et al., 2018; Oliveira-Brochado et al., 2017; Amornsriwatanakul et al., 2023; Eime et al., 2015; Wicker et al., 2013; Hallmann et al., 2012). We hypothesize that these factors are also likely to influence the frequency of TCC participation among adults. The independent variables span multiple domains, including demographic characteristics, family background, physical and mental health status, history of GSP, diversity of sport involvement, lifestyle habits, participation motivations, environmental conditions, and TCC-specific background. Detailed measurements and operationalization of these variables are presented in Table 1.

**Table 1:** Overview of the measurement of the independent variables.

Variables	Questions	Options	Variable categories
Gender	What is your gender?	Men or women	Demographic characteristics
Age	In which of the following categories does your age fall?	Young adults (18–45 years), middle-aged (45–60 years), and older adults (60 years and above)	Demographic characteristics
Education	What is your highest level of education?	High school or below, Associate degree, Bachelor’s degree, and Master’s degree or above	Demographic characteristics
The nature of labor	Which category does the nature of your work fall into?	Mental labor, manual labor, and combined mental and manual labor	Demographic characteristics
Immigration	Were you born outside of Zhengzhou?	Yes or no	Social background
Income	Which of the following ranges is your personal income?	Under 2,000 yuan, 2,000–5,000 yuan, 5,000–8,000 yuan, and over 8,000 yuan	Social background
Work hours	How many hours do you work on average per day?	Not working or retired, 8 h or less, 9–10 h, and Over 10 h	Social background
Types of work organizations	What Type of work organizations does your workplace belong to?	Unemployed, self-employed, private or foreign enterprises, state-owned enterprises, and government or administrative units	Social background
Children	Do you have minor children?	Yes or no	Family background
Household size	How many people are currently living in your home?	2 people or fewer, 3 people, 4–5 people, and 6 or more people	Family background
Health problems	Do you suffer from certain diseases?	Yes or no	Physical and mental health status
Health perception	Do you often feel physical discomfort?	Yes or no	Physical and mental health status
Mental problems	In the last year, have you experienced chronic anxiety or depression and used psychotropic medications? (such as tranquilizers or sleep aids)	Yes or no	Physical and mental health status
Smoker	Do you smoke?	Yes or no	Lifestyle habits
Alcohol	How many times a week do you drink alcohol on average?	No alcohol, 2 times or less per week, 3–4 times per week, and 5 times or more per week	Lifestyle habits
Watched competitions	Do you regularly watch martial arts competitions?	Yes or no	Lifestyle habits
Previous non-martial arts	Did you regularly participate in non-martial arts sports (i.e., basketball, badminton, etc.) before practicing TCC?	Yes or no	Sports participation history
Previous other martial arts	Did you regularly participate in other martial arts sports (i.e., boxing, taekwondo, etc.) before practicing TCC?	Yes or no	Sports participation history
Years of participation	What is your cumulative number of years practicing TCC?	Less than 1 year, 1–3 years, 4–10 years, and more than 10 years	Sports participation history
Awards	What is the overview of your tai chi competition awards?	“None,” “county level,” “city level” and “provincial level or higher.”	Sports participation history
High-intensity TCC	Do you currently practice types of high-intensity TCC?	Yes or no	Diversity of sports participation
Current non-martial	Do you currently participate in non-martial arts sports?	Yes or no	Diversity of sports participation
Current other martial arts	Do you currently practice other martial arts?	Yes or no	Diversity of sports participation
Health motivation	Do you participate in TCC for health?	Yes or no	Motivation to participate
Enjoyment motivation	Do you participate in TCC for enjoyment?	Yes or no	Motivation to participate
Weight loss motivation	Do you participate in TCC for weight loss?	Yes or no	Motivation to participate
Social interaction motivation	Do you participate in TCC for social interaction?	Yes or no	Motivation to participate
Competition preparation motivation	Do you participate in TCC for competition preparation?	Yes or no	Motivation to participate
Combat motivation	Do you participate in TCC for learning combat skills?	Yes or no	Motivation to participate
Alternate spaces	Do you have an alternative practice space near your residence other than parks?	Yes or no	Environment of participation
Indoor spaces	Do you have a suitable indoor practice space?	Yes or no	Environment of participation
Disturbances	Are you often disturbed (i.e., noise, pedestrians, etc.) when you participate in TCC at the park?	Yes or no	Environment of participation
Distance	How far do you live within walking distance of the park?	Less than 5 min, 6–10 min, 11–20 min, and more than 20 min	Environment of participation
Partners	How many people are usually practicing TCC with you at the same time?	No-one, 1–5 people, 6–10 people, and more than 10 people	TCC participation background
Investment	What is the cumulative amount you have invested in the sport of tai chi?	Less than 3,000 yuan, 3,000–6,000 yuan, 6,000–10,000 yuan, and more than 10,000 yuan	TCC participation background

### 2.3 Data analysis

All data analyses were conducted using IBM SPSS Statistics for Windows (Version 27.0; IBM Corp., Armonk, NY, USA). First, all variables from the 998 valid samples were coded to facilitate subsequent data processing and analysis. To examine the correlates of TCC participation frequency among Chinese adults, this study adopted a two-step modeling approach based on the analytical strategies proposed by Deelen et al. (2018) and Amornsriwatanakul et al. (2023).

In the first step, all independent variables were subjected to univariate analysis. Variables that showed a statistically significant association with the outcome variable—TCC participation frequency (*p* < 0.05)—were selected for inclusion in the subsequent ordinal regression models, while non- significant variables were excluded. This selection criterion was applied to the backward stepwise ordinal regression modeling. Given that the outcome variable is ordinal and the independent variables included both ordinal and nominal categorical variables, the nonparametric rank-sum test was used for nominal variables, and the Gamma test was applied for ordinal variables. Prior to regression modeling, Spearman correlation analysis was conducted on the screened variables to assess potential multicollinearity. No significant multicollinearity was found.

In the second step, all variables identified in the first step were entered into the initial ordinal regression model. Using the backward stepwise procedure, only those variables that remained significantly associated with the outcome variable (*p* < 0.05) were retained, while non- significant variables were removed in subsequent models. This process continued until the final model included only statistically significant predictors.

Additionally, each ordinal regression model was subjected to a test of the parallel lines assumption to ensure model validity (*p* > 0.05). Model fit was further evaluated by comparing the −2 Log Likelihood values across models to assess improvements in model performance.

## 3 Result

Our analysis was based on the 998 respondents (83. 2%) who completed all questionnaire items out of a total of 1, 200 surveyed individuals. Descriptive statistics for the overall sample are presented in Table 2, which also includes the results of the univariate analysis for TCC participation frequency, displaying only statistically significant coefficients. Table 3 presents the results of the backward stepwise ordinal regression analysis.

**Table 2:** Characteristics of the samples and the univariate analysis of correlates of TCC participation frequency.

Variables	Options	Total (*n* = 998)		1–2 times (*n* = 113) 11.3%		3–4 times (*n* = 292) 29.3%		5–6 times (*n* = 380) 38.1%		7 times and above (*n* = 213) 21.3%		*p*
		*n*	%	*n*	%	*n*	%	*n*	%	*n*	%	
Age	Young adults	233	23.3	67	59.3	119	40.8	35	9.2	12	5.6	0
	Middle-aged adults	463	46.4	19	16.8	127	43.5	187	49.2	130	61.0	
	Older adults	302	30.3	27	23.9	46	15.8	158	41.6	71	33.3	
Gender	Female	662	66.3	83	73.5	213	72.9	276	72.6	90	42.3	0
	Male	336	33.7	30	26.5	79	27.1	104	27.4	123	57.7	
Income	Under 2,000 yuan	109	10.9	11	9.7	32	11	42	11.1	24	11.3	0.923
	2,000–5,000 yuan	626	62.7	72	63.7	178	61	249	65.5	127	59.6	
	5,000–8,000 yuan	200	20	27	23.9	59	20.2	67	17.6	47	22.1	
	Over 8,000 yuan	63	6.3	3	2.7	23	7.9	22	5.8	15	7	
Education	High school or below	402	40.3	10	8.8	75	25.7	204	53.7	113	53.1	0
	Associate degree	442	44.3	69	61.1	144	49.3	142	37.4	87	40.8	
	Bachelor’s degree	133	13.3	34	30.1	59	20.2	30	7.9	10	4.7	
	Master’s degree or above	21	2.1	0	0	14	4.8	4	1.1	3	1.4	
Household size	2 people or fewer	49	4.9	10	8.8	11	3.8	24	6.3	4	1.9	0.843
	3 people	316	31.7	24	21.2	95	32.5	128	33.7	69	32.4	
	4–5 people	312	31.3	34	30.1	106	36.3	109	28.7	63	29.6	
	6 or more people	321	32.2	45	39.8	80	27.4	119	31.3	77	36.2	
Children	No	629	63	39	34.5	140	47.9	285	75	165	77.5	0
	Yes	369	37	74	65.5	152	52.1	95	25	48	22.5	
Types of work organizations	Unemployed	38	3.8	2	1.8	22	7.5	13	3.4	1	0.5	0
	Self-employed	98	9.8	12	10.6	25	8.6	36	9.5	25	11.7	
	Private or foreign enterprises	169	16.9	30	26.5	58	19.9	50	13.2	31	14.6	
	State-owned enterprises	416	41.7	41	36.3	113	38.7	167	43.9	95	44.6	
	Government or Administrative units	277	27.8	28	24.8	74	25.3	114	30.0	61	28.6	
Nature of labor	Mental labor	463	46.4	9	8	81	27.7	225	59.2	148	69.5	0
	Manual labor	295	29.6	71	62.8	136	46.6	59	15.5	29	13.6	
	Combined mental and manual labor	240	24	33	29.2	75	25.7	96	25.3	36	16.9	
Work hours	Not working or retired	597	59.8	41	36.3	173	59.2	269	70.8	114	53.5	0.019
	8 h or less	274	27.5	46	40.7	82	28.1	82	21.6	64	30	
	9–10 h	103	10.3	26	23	30	10.3	18	4.7	29	13.6	
	Over 10 h	24	2.4	0	0	7	2.4	11	2.9	6	2.8	
Smoker	No	901	90.3	99	87.6	273	93.5	346	91.1	183	85.9	0.096
	Yes	97	9.7	14	12.4	19	6.5	34	8.9	30	14.1	
Alcohol	No alcohol	775	77.7	76	67.3	246	84.2	315	82.9	138	64.8	0.034
	2 times or less per week	154	15.4	25	22.1	34	11.6	48	12.6	47	22.1	
	3–4 times per week	48	4.8	8	7.1	7	2.4	14	3.7	19	8.9	
	5 times or more per week	21	2.1	4	3.5	5	1.7	3	0.8	9	4.2	
Health problems	No	752	75.4	108	95.6	222	76	273	71.8	149	70	0
	Yes	246	24.6	5	4.4	70	24	107	28.2	64	30	
Health perception	No	458	45.9	45	39.8	128	43.8	170	44.7	115	54	0.012
	Yes	540	54.1	68	60.2	164	56.2	210	55.3	98	46	
Years of participation	Less than 1 year	194	19.4	39	34.5	88	30.1	50	13.2	17	8	0
	1–3 years	326	32.7	55	48.7	76	26	123	32.4	72	33.8	
	4–10 years	262	26.3	6	5.3	83	28.4	128	33.7	45	21.1	
	More than 10 years	216	21.6	13	11.5	45	15.4	79	20.8	79	37.1	
Investment (yuan)	Less than 3,000 yuan	400	40.1	111	98.2	207	70.9	67	17.6	15	7	0
	3,000–6,000 yuan	131	13.1	1	0.9	39	13.4	43	11.3	48	22.5	
	6,000–10,000 yuan	232	23.2	1	0.9	30	10.3	137	36.1	64	30	
	More than 10,000 yuan	235	23.5	0	0	16	5.5	133	35	86	40.4	
Migration	Yes	449	45	39	34.5	127	43.5	171	45	112	52.6	0.003
	No	549	55	74	65.5	165	56.5	209	55	101	47.4	
Alternate space	No	394	39.5	96	85	226	77.4	46	12.1	26	12.2	0
	Yes	604	60.5	17	15	66	22.6	334	87.9	187	87.8	
Distance	Less than 5 min	325	32.6	5	4.4	37	12.7	171	45	112	52.6	0
	6–10 min	355	35.6	5	4.4	75	25.7	185	48.7	90	42.3	
	11–20 min	174	17.4	43	38.1	109	37.3	15	3.9	7	3.3	
	More than 20 min	144	14.4	60	53.1	71	24.3	9	2.4	4	1.9	
High-intensity TCC	No	939	94.1	112	99.1	275	94.2	351	92.4	201	94.4	0.147
	Yes	59	5.9	1	0.9	17	5.8	29	7.6	12	5.6	
Competitions preparation	No	888	89	108	95.6	279	95.5	332	87.4	169	79.3	0
	Yes	110	11	5	4.4	13	4.5	48	12.6	44	20.7	
Watched competitions	No	382	38.3	34	30.1	135	46.2	152	40	61	28.6	0.035
	Yes	616	61.7	79	69.9	157	53.8	228	60	152	71.4	
Awards	None	375	37.6	87	77	195	66.8	61	16.1	32	15	0
	County level	188	18.8	8	7.1	23	7.9	103	27.1	54	25.4	
	City level	283	28.4	11	9.7	62	21.2	135	35.5	75	35.2	
	Provincial level or higher	152	15.2	7	6.2	12	4.1	81	21.3	52	24.4	
Previous non-martial arts	No	662	66.3	83	73.5	200	68.5	243	63.9	136	63.8	0.051
	Yes	336	33.7	30	26.5	92	31.5	137	36.1	77	36.2	
Previous other martial arts	No	926	92.8	105	92.9	267	91.4	356	93.7	198	93	0.548
	Yes	72	7.2	8	7.1	25	8.6	24	6.3	15	7	
Current non-martial arts	No	694	69.5	81	71.7	214	73.3	259	68.2	140	65.7	0.064
	Yes	304	30.5	32	28.3	78	26.7	121	31.8	73	34.3	
Current other martial arts	No	956	95.8	113	100	283	96.9	361	95	199	93.4	0.003
	Yes	42	4.2	0	0	9	3.1	19	5	14	6.6	
Combat motivation	No	978	98	113	100	291	99.6	370	97.4	204	95.8	0
	Yes	20	2	0	0	1	0.4	10	2.6	9	4.2	
Health motivation	No	161	16.1	97	85.8	32	11	18	4.7	14	6.6	0
	Yes	837	83.9	16	14.2	260	89	362	95.3	199	93.4	
Social interaction Motivation	No	785	78.7	97	85.8	234	80.1	280	73.7	174	81.7	0.272
	Yes	213	21.3	16	14.2	58	19.9	100	26.3	39	18.3	
Weight loss motivation	No	774	77.6	81	71.7	232	79.5	298	78.4	163	76.5	0.769
	Yes	224	22.4	32	28.3	60	20.5	82	21.6	50	23.5	
Enjoyment motivation	No	275	27.6	36	31.9	82	28.1	106	27.9	51	23.9	0.162
	Yes	723	72.4	77	68.1	210	71.9	274	72.1	162	76.1	
Mental problems	No	851	85.3	77	68.1	234	80.1	347	91.3	193	90.6	0
	Yes	147	14.7	36	31.9	58	19.9	33	8.7	20	9.4	
Partners	No-one	131	13.1	40	35.4	74	25.3	10	2.6	7	3.3	0
	1–5 people	255	25.6	52	46	144	49.3	36	9.5	23	10.8	
	6–10 people	170	17	4	3.5	19	6.5	97	25.5	50	23.5	
	More than 10 people	442	44.3	17	15	55	18.8	237	62.4	133	62.4	
Disturbances	No	613	61.4	63	55.8	182	62.3	237	62.4	131	61.5	0.536
	Yes	385	38.6	50	44.2	110	37.7	143	37.6	82	38.5	
Indoor space	No	787	78.9	93	82.3	242	82.9	290	76.3	162	76.1	0.028
	Yes	211	21.1	20	17.7	50	17.1	90	23.7	51	23.9	

Percentages in each group may not total 100 due to rounding.

**Table 3:** Backward stepwise ordered regression model of the correlates of TCC participation frequency.

	Model 1			Model 2		
	*β*	SE	*p*	*β*	SE	*p*
Outcome variable						
Frequency (7 times and above = ref)						
1–2 times	1.781	0.985	0.071	2.36	0.399	0
3–4 times	6.623	1.031	0	7.107	0.495	0
5–6 times	10.1	1.06	0	10.482	0.554	0
Independent variables						
Age (older adults = ref)						
Young adults	−0.627	0.273	0.022	−0.481	0.225	0.033
Middle-aged adults	0.426	0.183	0.02	0.356	0.162	0.028
Gender (Female = ref)						
Male	0.896	0.168	0	1.009	0.151	0
Nature of labor (combined mental and manual labor = ref)						
Mental labor	0.9	0.182	0	0.875	0.176	0
Manual labor	0.157	0.207	0.448	0.111	0.202	0.584
Investment (less than 3,000 yuan = ref)						
3,000–6,000 yuan	2.044	0.259	0	1.982	0.25	0
6,000–10,000 yuan	1.41	0.234	0	1.406	0.228	0
More than 10,000 yuan	1.874	0.234	0	1.955	0.227	0
Migration (No = ref)						
Yes	−0.292	0.145	0.044	−0.307	0.141	0.029
Alternate space (No = ref)						
Yes	0.978	0.223	0	0.9	0.208	0
Distance (more than 20 min = ref)						
Less than 5 min	2.16	0.308	0	2.192	0.299	0
6–10 min	1.987	0.296	0	1.979	0.287	0
11–20 min	1.194	0.277	0	1.057	0.271	0
Awards (none = ref)						
County level	0.688	0.23	0.003	0.709	0.221	0.001
City level	0.722	0.227	0.001	0.797	0.203	0
Provincial level or higher	1.059	0.278	0	1.163	0.242	0
Health motivation (No = ref)						
Yes	2.299	0.236	0	2.287	0.23	0
Mental problems (No = ref)						
Yes	−0.493	0.213	0.021	−0.476	0.208	0.022
Partners (No-one = ref)						
1–5 people	0.239	0.262	0.361	0.166	0.255	0.515
6–10 people	1.277	0.303	0	1.235	0.297	0
More than 10 people	1.059	0.28	0	1.081	0.274	0
Health problems (No = ref)						
Yes	0.414	0.193	0.032	0.462	0.174	0.008
Education (Master’s degree or above = ref)				Exc		
High school or below	0.08	0.531	0.88			
Associate degree	0.134	0.523	0.797			
Bachelor’s degree	−0.568	0.545	0.298			
Years of participation (less than 1 year = ref)				Exc		
1–3 years	0.15	0.212	0.48			
4–10 years	−0.062	0.248	0.802			
More than 10 years	0.463	0.272	0.089			
Children (No = ref)				Exc		
Yes	−0.269	0.164	0.101			
Types of work organizations (government or administrative units = ref)				Exc		
Unemployed	0.215	0.423	0.258			
Self-employed	0.197	0.281	0.491			
Private or foreign enterprises	0.138	0.233	0.352			
State-owned enterprises	−0.075	0.179	0.176			
Work hours (Over 10 h = ref)				Exc		
Not working or retired	−0.258	0.475	0.587			
8 h or less	−0.305	0.473	0.519			
9–10 h	0.272	0.513	0.596			
Alcohol (5 times or more per week = ref)				Exc		
No alcohol	−0.178	0.539	0.74			
2 times or less per week	0.117	0.551	0.833			
3–4 times per week	0.155	0.631	0.807			
Health perception (No = ref)				Exc		
Yes	−0.287	0.147	0.051			
Competitions preparation (No = ref)				Exc		
Yes	0.095	0.241	0.691			
Watched competitions (No = ref)				Exc		
Yes	−0.08	0.151	0.596			
Current other martial arts (No = ref)				Exc		
Yes	−0.122	0.356	0.731			
Combat motivation (No = ref)				Exc		
Yes	0.326	0.536	0.543			
Indoor space (No = ref)				Exc		
Yes	−0.05	0.178	0.777			
Model fit information						
Nagelkerke *R*^2^	0.738			0.726		
P model	0			0		
Chi-Square	1149.36			1114.721		
−2 Log Likelihood (general)	1452.469			1422.89		
Test of Parallel Lines						
Chi-square	55.824c			20.563c		
*p*-value	0.998			0.999		

95%CI.

According to Table 2, the final sample included 998 respondents. Among them, middle-aged adults accounted for the largest proportion (46.4%), followed by older adults (30.3%) and young adults (23.3%). In terms of gender distribution, 66.3% of the respondents were female, nearly twice the proportion of males (33.7%). More than half of the participants practiced TCC relatively frequently, with 59.4% reporting participation five or more times per week. Regarding session duration, the most common category was 30–60 min, accounting for 45.9% of the sample, followed by 60–90 min (37.1%). In terms of income, over half of the respondents (62.7%) reported monthly earnings between 2,000 yuan and 5,000 yuan, which aligns closely with the average monthly income of 3,649 yuan for Zhengzhou residents in 2023 (Zhengzhou Statistics Bureau, 2024). The majority of respondents lived within a 10-min walking distance to their practice site, representing 68.2% of the sample. In terms of motivation, health improvement was the most frequently reported reason for participating in TCC (83.9%), followed by simple enjoyment of the activity (72.4%). Additional details for all variables are provided in Table 2.

Table 2 also presents the results of the univariate analysis of correlates of TCC participation frequency, showing only significant coefficients. The univariate analysis, using the rank sum and gamma tests, revealed that 25 out of 36 independent variables were significantly correlated with TCC participation frequency. These 25 variables were included in the subsequent ordered regression analysis. In the univariate analysis, the following variables had coefficients greater than 0.05 (*p* > 0.05) and were excluded from subsequent ordered regression analyses: disturbances (*p* = 0.536), enjoyment motivation (*p* = 0.162), weight loss motivation (*p* = 0.769), social interaction motivation (*p* = 0.272), current non-martial arts (*p* = 0.064), previous other martial arts (*p* = 0.548), previous non-martial arts (*p* = 0.051), high-intensity TCC (*p* = 0.147), smoker (*p* = 0.096), household size (*p* = 0.843), and income (*p* = 0.923).

Table 3 presents the results of the ordinal regression analysis examining the correlates of TCC participation frequency among Chinese adults. The model outputs include statistical significance levels, regression coefficients (*β*), and standard errors. The test of parallel lines confirmed the validity of both models (*p* > 0.05), indicating that the proportional odds assumption holds. The −2 Log Likelihood values further suggest improved model fit over iterations: the final model achieved a lower −2 log likelihood (1422.89) compared to the initial model (1452.469), indicating a better-fitting model. In the final model, age showed significant associations with participation frequency. Compared to older adults, young adults (*p* = 0.033, *β* = −0.481) reported significantly lower participation, while middle-aged adults (*p* = 0.028, *β* = 0.356) had significantly higher participation, making them the most frequent participants among all adult age groups. Regarding gender, males (*p* < 0.001, *β* = 1.009) participated more frequently than females. In terms of occupational type, individuals engaged in mental labor (*p* < 0.001, *β* = 0.875) reported higher participation frequencies. Financial investment in TCC practice was also positively associated with participation frequency. Compared to those who invested less than 3,000 yuan, individuals who invested 3,000 yuan–6,000 yuan (*p* < 0.001, *β* = 1.982), 6,000 yuan −10,000 yuan (*p* < 0.001, *β* = 1.406), and more than 10,000 yuan (*p* < 0.001, *β* = 1.955) participated more frequently. Migrants (*p* = 0.029, *β* = −0.307) reported significantly lower participation compared to local residents. The availability of an alternative practice space near the residence (*p* < 0.001, *β* = 0.900) was positively associated with higher participation frequency. Walking distance to the regular practice site (park) was another key factor. Compared to participants with walking distances of more than 20 min, those with distances of less than 5 min (*p* < 0.001, *β* = 2.192), 6–10 min (*p* < 0.001, *β* = 1.979), and 11–20 min (*p* < 0.001, *β* = 1.057) participated more frequently. The decreasing walking distance was associated with increasing β values, indicating that proximity to the practice location is a strong facilitator of higher participation frequency. Award history also showed a positive correlation with frequency. Compared to those who had never received awards, participants with county-level (*p* = 0.001, *β* = 0.709), city-level (*p* < 0.001, *β* = 0.797), and provincial-level or higher awards (*p* < 0.001, *β* = 1.163) were significantly more likely to engage frequently, and the *β* values increased with award level, suggesting a dose–response relationship. Health motivation (*p* < 0.001, *β* = 2.287) was strongly associated with increased participation. Conversely, individuals with mental health problems (*p* = 0.022, *β* = −0.476) such as anxiety and insomnia participated significantly less frequently. The number of practice companions also mattered. Participants practicing in groups of 6–10 (*p* < 0.001, *β* = 1.235) and more than 10 (*p* < 0.001, *β* = 1.081) reported significantly higher participation frequencies compared to those who practiced alone. Interestingly, participants with physical health problems (*p* = 0.008, *β* = 0.462) reported higher participation frequencies than those without, possibly due to health-motivated engagement in TCC. Other variables were not significantly associated with participation frequency (*p* > 0.05).

## 4 Discussion

This study utilized a representative sample collected from public parks in Zhengzhou City, China, to examine the correlates of TCC participation frequency among Chinese adults (aged 18 and above). Findings showed that many of the previously identified potential factors affecting GSP were also significantly associated with the frequency of TCC practices, while some results differed from those reported in the GSP study. Specifically, the variables found to be significantly associated with TCC participation frequency included age, gender, type of work, financial investment, migration status, availability of alternative practice spaces, walking distance from home to practice parks, competition awards, health-related motivation, mental health problems, number of practice companions, and physical health issues. In contrast, the remaining independent variables showed no significant associations.

Among these findings, the most notable discrepancy compared to GSP literature lies in the effect of age, highlighting the unique patterns of TCC participation across different demographic groups. This study identified a significant association between age and the frequency of TCC participation, however, the effect size notably differ from much of the existing literature on GSP (Zasimova, 2022; Borgers et al., 2016; Oliveira-Brochado et al., 2017). The results reveal that middle-aged adults exhibit the highest frequency of TCC practice, followed by older adults, while young adults participate the least. This is a key finding of the study, indicating that within the adult population, young individuals are the least engaged in TCC, whereas middle-aged individuals are the most active. The lower participation frequency among older adults may be attributed to physical limitations due to age-related functional decline (Chad et al., 2005; Downward and Rasciute, 2010; Farrell and Shields, 2002; Seippel and Bergesen Dalen, 2024; Ruseski and Maresova, 2014). In contrast, the low frequency among young adults may reflect differences in sport preferences across age groups. Prior research has shown that various types of physical activity appeal differently to different age cohorts (Wicker et al., 2013). Young adults tend to favor strength- based and outwardly expressive sports such as basketball or football (Seippel and Bergesen Dalen, 2024), whereas TCC is characterized by gentle movements and a strong emphasis on wellness. Given their generally good physical condition, young adults may feel less urgency to engage in health-preserving activities like TCC, reducing its attractiveness and their likelihood of participation.

Furthermore, this study found that middle-aged adults were the most frequent participants in TCC, contradicting some earlier studies suggesting that middle-aged individuals participate the least in physical activity (Zasimova, 2022; Ruseski et al., 2011). Previous literature has attributed higher participation rates among young people to stronger interest, and among older adults to greater time availability. However, our findings suggest that despite having more leisure time, older adults may prioritize health to a lesser extent than middle-aged adults. Physiological research indicates that physical decline is not a linear process but tends to accelerate around midlife, particularly near age 44–45 (Shen et al., 2024). This sharp decline may trigger increased health awareness and concern among middle-aged individuals. Additionally, compared to older adults, middle-aged individuals are more likely to possess the physical capacity to improve their health through exercise. The health and rehabilitation benefits of TCC have been well documented (Wang et al., 2014; Zheng et al., 2015), and the practice has been officially recommended in the National Fitness Guidelines (2017) issued by the General Administration of Sport of the People’s Republic of China (2017). This alignment between TCC’s health benefits and the health concerns of middle-aged adults makes them particularly inclined toward frequent participation. Their strong health motivation, combined with sufficient physical ability, positions them as the most frequent practitioners of TCC.

In terms of gender, the study found that men participated in TCC more frequently than women, a result consistent with much of the existing literature on GSP (Oliveira-Brochado et al., 2017; Downward et al., 2011; Downward et al., 2014; Mutter and Pawlowski, 2014). However, descriptive statistics revealed that women made up nearly twice the number of male respondents in the sample. Given the random sampling approach, this suggests that, in absolute terms, more women than men may be practicing TCC in Zhengzhou. This phenomenon may be attributed to gender differences in exercise preferences and participation intentions. Research has shown that men are generally more inclined toward strength training, whereas women tend to prefer aerobic and flexibility-oriented activities (Nuzzo and Deaner, 2023). For instance, yoga, which emphasizes slow, flowing, and coordinated movements, is often rated as more appealing by women (Zou et al., 2018). Given the similarities between TCC and yoga in terms of movement characteristics, TCC may similarly appeal to women. Nevertheless, women’s participation frequency remained significantly lower than that of men. Further analysis suggests that sociocultural factors may constrain women ‘s level of engagement (Downward et al., 2012; Charway and Strandbu, 2024; Muñiz et al., 2014). Studies have indicated that patriarchal structures limit women’s opportunities to engage in sports and physical activity. Marriage and childbirth have been shown to significantly reduce participation rates, with a greater impact on women. Additionally, cultural barriers— such as household responsibilities and traditional gender role expectations—further restrict women’s opportunities to exercise. Therefore, improving women’s participation in TCC requires not only individual-level interventions but also broader sociocultural shifts. Promoting gender equality, reshaping societal perceptions of gender roles, and creating a supportive environment are essential steps toward enhancing women’s participation in physical activity and achieving broader public health goals.

The findings of this study regarding exercise history and participation environment align closely with existing research on GSP (Zasimova, 2022; Downward et al., 2014; Downward and Riordan, 2007; An and Zheng, 2014; Atkinson et al., 2005; Owen et al., 2004). Specifically, award experience serves as an indicator of a positive exercise history, and previous studies have shown that such positive experiences significantly enhance the likelihood of continued participation (Downward and Riordan, 2007). Furthermore, based on B.F. Skinner’s operant conditioning theory (Skinner, 1938), positive reinforcement is effective in increasing the frequency of specific behaviors. In this context, receiving awards functions as a form of positive reinforcement, which can strongly motivate individuals to engage in TCC more consistently and at a higher frequency. Goal-setting theory is also significant in explaining why TCC practitioners who have received high-level awards tend to engage more frequently. This theory posits that more specific and challenging goals elicit higher levels of effort, greater persistence, and improved performance (Locke and Latham, 2019). Consequently, TCC participants who have earned advanced awards typically undergo systematic, goal-directed training, demonstrating greater goal clarity, stronger goal commitment, and higher self-efficacy. These psychological mechanisms make them more conscious of maintaining or elevating their practice levels, setting higher participation frequency goals, and even exhibiting greater perseverance in overcoming situational obstacles (such as weather or time conflicts).

At the environmental level, this study found that shorter walking distances to practice venues and the availability of alternative practice spaces were significantly positively associated with the frequency of TCC participation. A substantial body of literature has established that the accessibility and availability of sports facilities are key determinants of physical activity engagement (Zasimova, 2022; Downward et al., 2014; An and Zheng, 2014; Atkinson et al., 2005; Owen et al., 2004). Shorter walking distances reduce travel time and opportunity costs, thereby enhancing both the willingness and actual rate of participation. Additionally, the presence of alternative venues ensures continuity and stability in practice, particularly when the primary location is unavailable. Goal-setting theory also supports these findings to some extent. When participants have specific participation goals regarding the frequency of TCC practice, favorable situational factors—such as high availability and accessibility of practice venues—facilitate goal attainment (Locke and Latham, 2019), thereby ensuring participation rates.

This study also found no significant association between income level and TCC participation frequency, a finding consistent with previous research on GSP (Hallmann et al., 2012; Downward and Riordan, 2007; Lera-López and Rapún-Gárate, 2011). However, personal financial investment in TCC was significantly positively associated with participation frequency. As highlighted by Downward et al., individual spending and investment in physical activity can effectively promote more frequent engagement (Downward and Riordan, 2007). Economic costs associated with participation are often cited as substantial barriers (Eime et al., 2013; Boone-Heinonen et al., 2011; Costello et al., 2011); thus, personal financial investment may help to overcome these constraints and encourage higher levels of participation.

Health motivation was also positively associated with participation frequency, aligning with numerous studies in the field (Oliveira-Brochado et al., 2017; Downward et al., 2014; Muñiz et al., 2014; Lera-López and Rapún-Gárate, 2011; Teixeira et al., 2012; García et al., 2011). Extensive research has demonstrated that TCC significantly improves physical health, directly addressing individuals’ health- related needs (Jiménez-Martín et al., 2013; Yang et al., 2015; Wang et al., 2014; Huston and McFarlane, 2016; Chang et al., 2024). As such, health motivation emerges as a key factor driving high-frequency participation in TCC. Goal-setting theory also provides a compelling explanation for this outcome: individuals who establish clear, valuable, and meaningful goals tend to demonstrate greater motivation to act (Locke and Latham, 2019). Health motivation can be understood as a strategic goal—that is, individuals aim to maintain or improve their physical health through regular TCC practice. They recognize that practicing TCC is essential for their well-being. Simultaneously, their goals carry high value because health objectives hold long-term significance for them.

This study identified a significant negative association between mental health problems and the frequency of TCC participation, a finding consistent with the conclusions reported by Oliveira-Brochado et al. (2017). Psychological disorders are often accompanied by symptoms such as insomnia, early awakening, and insufficient sleep (Laskemoen et al., 2019), which can lead to persistent fatigue and substantially reduce individuals’ willingness and capacity to engage in physical activity. Moreover, mental health issues may cause diminished interest and reduced self- confidence—psychological traits that further suppress motivation for exercise (Xia et al., 2025). Collectively, these factors negatively influence the frequency of TCC participation among affected individuals.

The number of partners was significantly positively correlated with participation frequency, consistent with findings from numerous studies on GSP correlates (Crossman et al., 2024; Brinkley et al., 2017; Andersen et al., 2019). The study found that participation frequency significantly increased when the number of TCC partners exceeded six members, compared to practicing alone. Practicing TCC with multiple participants at the same time is a team activity, offering exercise partners for participants. Literature indicates that exercise partners are key factors in promoting physical activity participation among adults (Crossman et al., 2024). Team sports significantly enhance both physical and mental well- being (Brinkley et al., 2017). Moreover, evidence suggests that the social nature of team sports encourages participation (Andersen et al., 2019). These factors may explain why having partners is positively associated with participation frequency.

This study found that participants with health problems reported a higher frequency of TCC participation compared to those without such conditions. This result aligns with findings from certain prior studies on GSP (Muñiz et al., 2014; García et al., 2011). One possible explanation is that individuals with health concerns may be motivated to engage in exercise as a means of improving their health or may do so following medical advice. Research indicates that energy mobilization plays a dominant role in intense exercise forms. For instance, the cardiovascular parameter changes of sprinters and long-distance runners differ entirely. Sprinters’ heart rates reach 90 to 120 beats per minute even before the race begins, while long-distance runners experience little to no change. This means the body’s energy mobilization occurs before the actual physical demands of the sport, signifying anticipated behavioral adaptation (Schumann et al., 2022). Individuals with health issues often exhibit diminished bodily mobilization capacity. TCC’s slow movements demand minimal physiological mobilization, making it particularly suitable for such populations. Furthermore, intense exercise requires greater bodily mobilization, consuming significant energy and physical effort, with recovery occurring afterward. TCC, however, balances mobilization and recovery throughout its practice. In TCC, these two processes occur almost simultaneously. Each TCC movement demands focused, coordinated, and controlled exertion—requiring mobilization—yet this physical effort is swiftly counterbalanced by rhythmic breathing and sensory calm. This rhythm of activation and renewal creates what is termed dynamic equilibrium. The body’s physiological systems remain active yet unstrained; attention is engaged but swiftly replenished, preventing exhaustion. From this perspective, TCC practice allows practitioners to simultaneously experience both invigoration and restoration. This unique characteristic of TCC makes it particularly well-suited for participants with health concerns. However, the finding that participants with health issues engaged more frequently should still be interpreted with caution. Although the variable” perceived health status” was not retained in the final model, it demonstrated marginal significance in the initial model (*p* = 0.051) with a negative regression coefficient (*β* = −0.287). This suggests that when individuals experience noticeable physical discomfort, their participation frequency may decline. Previous research has indicated that as health deteriorates, engagement in physical activity tends to diminish (Eberth and Smith, 2010), likely due to physiological limitations and discomfort that inhibit participation. Therefore, individuals with health issues who do not yet experience severe symptoms may maintain a higher level of participation, whereas those with advanced or debilitating conditions may reduce their engagement. These findings imply a potential nonlinear relationship between health status and participation frequency in TCC, highlighting the need for further research to explore the underlying mechanisms.

The findings regarding the nature of labor align with several studies (Boutelle et al., 2000; Burton and Turrell, 2000). This may be due to the fact that mental laborers, typically white-collar workers, belong to higher social classes and often engage in work that lacks physical activity. In contrast, manual and brain- body laborers expend significant physical energy at work, leaving them with insufficient stamina to engage in physical activity during leisure time. Furthermore, manual laborers, often blue-collar workers, may have lower social status and may lack awareness of the health benefits of exercises like TCC. Additionally, the cost and time commitment of exercise may act as barriers to participation for this group, further reducing their participation frequency.

Migrants, defined as TCC participants born outside of Zhengzhou, participated less frequently than non-migrants. This finding aligns with research on GSP, which identifies immigration as a negative factor influencing participation behavior (Wicker et al., 2013; Hallmann et al., 2012). Zhengzhou City, the capital of Henan Province, is the only city in the province with a net population inflow, attracting many migrants from other cities and leading the province in economic development (Henan Provincial Bureau of Statistics, 2022; Henan Provincial Bureau of Statistics, 2024). This significant economic gap means that foreign-born residents in Zhengzhou often face greater financial pressures, such as mortgages and car loans, compared to locals, leading them to focus more on earning a living. In contrast, locally born residents in Zhengzhou often have a lighter financial burden, as many own ancestral homes or inherit property, thus avoiding mortgage pressures. Additionally, locally born residents often possess economic, cultural, and personal advantages, allowing them to integrate more easily into local social networks. These objective facts provide them with the conditions to participate in TCC more frequently, creating variability in participation rates between migrants and non-migrants.

## 5 Conclusion

This study provides an in-depth examination of the correlates of TCC participation frequency among Chinese adults. While many of these correlates align with those identified in GSP research, notable differences were also observed. Based on the findings, future promotion strategies aimed at increasing participation frequency in TCC should place greater emphasis on targeting specific subgroups—particularly mental laborers, males, middle-aged adults, individuals with mild health issues, and local residents—by highlighting the health benefits of TCC. Effective intervention measures may include improving the accessibility and availability of practice spaces, encouraging greater financial investment in TCC, increasing opportunities for competition and awards, fostering social practice groups, and addressing mental health issues. Additionally, efforts to reshape gender-related sociocultural perceptions will be critical in promoting more equitable and widespread participation in TCC.

Current research on TCC has predominantly focused on its health benefits, while limited attention has been given to the behavioral factors influencing participation. This study addresses this gap by employing backward stepwise ordinal regression to analyze the correlates of TCC participation frequency. Notably, the study reveals innovative differences from existing research on GSP, particularly with respect to age-related patterns. These findings offer a novel perspective and empirical evidence, providing a theoretical foundation for the development of effective intervention strategies and advancing research in the field of TCC participation.

Despite offering valuable insights into the frequency of TCC participation, this study has several limitations. First, as a cross-sectional study, it does not allow for causal inference and can only describe associations between variables. Second, the data were collected through self-reported questionnaires, which may be subject to response bias, such as overestimation or underestimation by participants, potentially affecting data accuracy. Moreover, the study exclusively focused on individuals who already participate in TCC, without including non-participants; this may limit the comparability of findings with studies that include broader populations. Lastly, the sample was primarily drawn from urban park settings, excluding rural areas and specialized environments such as martial arts schools or training centers, which may affect the generalizability of the results to a wider population.

To develop more effective TCC promotion strategies or interventions, future research should explore causal relationships between independent variables and TCC participation behaviors through longitudinal or intervention studies. Expanding the sample sources to include more diverse settings and populations is also crucial for enhancing the generalizability and representativeness of the study. Future studies should use more comprehensive analytical methods, incorporate additional levels of independent variables, and apply advanced models (e.g., ensemble learning algorithms) to explore the correlates of TCC participation more thoroughly and accurately. Additionally, future studies could examine other TCC participation behaviors (e.g., whether the recommended dose was achieved, time spent, and exercise intensity) to gain a more comprehensive understanding of the factors influencing participation.

## Data Availability

The original contributions presented in the study are included in the article/supplementary material, further inquiries can be directed to the corresponding author.
